# Pathophysiology of Red Blood Cell Dysfunction in Diabetes and Its Complications

**DOI:** 10.3390/pathophysiology30030026

**Published:** 2023-08-02

**Authors:** Alyssa Williams, Rosi Bissinger, Hala Shamaa, Shivani Patel, Lavern Bourne, Ferruh Artunc, Syed M. Qadri

**Affiliations:** 1 Faculty of Science, Ontario Tech University, Oshawa, ON L1G 0C5, Canada; 2 School of Biomedical Engineering, McMaster University, Hamilton, ON L8S 4M1, Canada; 3 Division of Endocrinology, Diabetology and Nephrology, Department of Internal Medicine, University Hospital Tübingen, 72076 Tübingen, Germany; 4 Faculty of Health Sciences, Ontario Tech University, Oshawa, ON L1G 0C5, Canada; 5 Institute of Diabetes Research and Metabolic Diseases of the Helmholtz Center Munich at the University of Tübingen, 72076 Tübingen, Germany; 6 German Center for Diabetes Research at the University of Tübingen, 72076 Tübingen, Germany

**Keywords:** diabetes, red blood cells, metabolism, anemia, cell death, deformability, complications, microcirculation, thrombosis

## Abstract

Diabetes Mellitus (DM) is a complex metabolic disorder associated with multiple microvascular complications leading to nephropathy, retinopathy, and neuropathy. Mounting evidence suggests that red blood cell (RBC) alterations are both a cause and consequence of disturbances related to DM-associated complications. Importantly, a significant proportion of DM patients develop varying degrees of anemia of confounding etiology, leading to increased morbidity. In chronic hyperglycemia, RBCs display morphological, enzymatic, and biophysical changes, which in turn prime them for swift phagocytic clearance from circulation. A multitude of endogenous factors, such as oxidative and dicarbonyl stress, uremic toxins, extracellular hypertonicity, sorbitol accumulation, and deranged nitric oxide metabolism, have been implicated in pathological RBC changes in DM. This review collates clinical laboratory findings of changes in hematology indices in DM patients and discusses recent reports on the putative mechanisms underpinning shortened RBC survival and disturbed cell membrane architecture within the diabetic milieu. Specifically, RBC cell death signaling, RBC metabolism, procoagulant RBC phenotype, RBC-triggered endothelial cell dysfunction, and changes in RBC deformability and aggregation in the context of DM are discussed. Understanding the mechanisms of RBC alterations in DM provides valuable insights into the clinical significance of the crosstalk between RBCs and microangiopathy in DM.

## 1. Introduction

Diabetes mellitus (DM), characterized by chronic elevation of blood glucose levels, is one of the leading causes of global mortality rates [1,2]. The prevalence of DM has reportedly increased by 129.7% from 1990 to 2017 and is expected to rise without the use of effective interventions [2]. It is estimated that by the year 2045, there will be a global increase to 700 million adults living with DM [3]. Type 1 diabetes (T1D), type 2 diabetes (T2D), and other DM subtypes have a substantial economic burden and are a major public health concern [4]. T1D is characterized by the destruction of pancreatic beta cells, resulting in a lifelong dependence on exogenous insulin. In patients with T2D, the insulin produced by the pancreas is not used effectively by the body [5,6]. T2D has a multifactorial etiology with factors including obesity, family history, and age [5]. While the incidence rates of diabetes have been relatively stable, there has been an increased prevalence of T2D in children and youth over the last two decades [7].

In DM, chronic hyperglycemia can lead to serious life-threatening complications and exacerbate damage done to various organs, including the kidneys, nervous system, and cardiovascular system [5,8]. Beyond this, alterations in various hematological parameters reflecting the function, structure, and metabolism of red blood cells (RBCs), white blood cells (WBCs), and platelets are commonly encountered during the clinical course of this condition [9,10,11]. In conjunction with chronic inflammation, oxidative stress, and endothelial cell (EC) dysfunction, the changes in hematological indices are believed to contribute to the pathophysiology of imbalanced cardiovascular and renal function, as well as inflammatory sequalae in DM patients [12,13]. Pathological alterations in RBC morphology and functions related to chronic hyperglycemia not only mechanistically underpin DM complications but can also be triggered by multisystemic changes such as the accumulation of toxins [14] and altered cellular signaling [15]. Chronic hyperglycemia stimulates redox imbalances [16], disturbs normal RBC cell membrane architecture [17], and alters the expression levels of various membrane transporters [18] alongside activities of the Na^2+^/K^+^ pump [19], Ca^2+^ ATPase [20], and acetylcholinesterase [21]. 

RBC dysfunction may lead to deranged tissue oxygenation through a variety of mechanisms. This includes impaired microcirculation and biochemical changes such as increased glycosylation of RBC 2,3-bisphosphoglycerate binding sites [22]. RBCs in DM patients further display disturbances in their biophysical properties contributing to changes in normal rheology and hemodynamics [23]. Collectively, these RBCs contribute to RBC dysfunction, reducing their lifespan in circulation and leading to anemia and the development of various DM-associated complications. 

Anemia is a frequently encountered clinical finding in DM patients [24,25]. Anemia, clinically defined as hemoglobin levels < 13.0 g/dL in men and <12.0 g/dL in women [26], is often neglected in DM patients, and when left untreated, it may increase the risk of the development and progression of various clinical complications. This review highlights the mechanisms of RBC dysfunction in DM, which underpin the pathophysiology of anemia, impaired microcirculation, and a procoagulant state in DM patients. 

## 2. Epidemiology and Subtypes of Anemia in Diabetes

Anemia is the most prominent clinical manifestation of RBC dysfunction in diabetes [27]. Past studies have shown that DM patients are at a higher risk of developing anemia as compared to non-diabetics [28,29]. However, anemia in DM patients is largely unrecognized, undetected, and untreated [30]. Anemia is widely (or historically) considered to be an endpoint of a long-term process involving the initiation of vascular damage in DM patients [31]. Importantly, anemia may be significant in determining the outcome of comorbid vascular disease in DM patients [32]. Along these lines, reduced hemoglobin levels could potentially identify DM patients at increased risk of hospitalization and mortality [33]. The prevalence of anemia in DM patients (both types 1 and 2) has been reported to display ethnic and geographic variations, ranging between 14% and 45%, among different populations worldwide [24,31,32,34,35,36]. The insidious development of anemia in DM patients was also shown to be more frequent in men and women older than 60 years of age [31]. The reduction in hemoglobin levels showed a significant correlation with both age at baseline and age at DM onset on univariate analysis [31]. A positive correlation was shown between the degree of anemia and glycated hemoglobin (HbA1c) levels in DM patients, supporting the view that poorly controlled DM promotes anemia [37].

Anemia is a complex and multifactorial disease that is diagnosed using RBC features. In addition to hemoglobin parameters, mean cell volume (MCV) is used to distinguish between microcytic, normocytic, and macrocytic anemia in DM patients [38]. Antwi-Bafour et al. studied patients with T2D and reported diagnoses of normochromic normocytic anemia (73.8%), hypochromic microcytic anemia (19.1%), and normochromic macrocytic anemia (7.1%) [37]. In a cross-sectional study, Hosseini et al. reported 30.4% of T2D patients with concurrent anemia; 15.1% of the T2D patients were diagnosed with normochromic normocytic, 14.4% with hypochromic microcytic, and 1% with macrocytic anemias [39]. Piñero-Piloña et al. found some patients with new-onset DM to have mild normochromic normocytic anemia, where the improvement in glycemic control tended to normalize hemoglobin levels [40]. In addition to the widely reported anisocytosis in RBCs of DM patients, poikilocytosis of RBCs is also known to be a common laboratory finding [41]. 

RBC membranes of DM patients show a wide array of changes. RBCs from patients with T2D were shown to have marked elevation in the cholesterol content of their cell membranes [42]. Atomic force and scanning electron microscopy techniques have provided robust evidence of the ultrastructural alterations in the diameter, axial ratio, and concave depth in RBCs from DM patients [17,43]. 

Clinical laboratory parameters related to RBC changes in DM gleaned from a complete blood count (CBC) using an automated hematology analyzer are a cornerstone in the diagnosis of anemia [44]. While HbA1c levels provide useful information on long-term glycemic control, it is not an indicator of RBC dysfunction per se. DM-associated anemia is primarily diagnosed based on changes in RBC count and hemoglobin, with alterations in hematocrit levels also considered [27]. A growing body of evidence suggests that changes in RBC distribution width (RDW), an index of heterogeneity in circulating RBC size, potentially serves as a prognostic marker to gauge DM complications and mortality risk [45,46]. Previous studies have analyzed RDW changes in DM in terms of either RDW-Standard Deviation (SD) or RDW-Coefficient of Variation (CV). A significantly increased risk of developing DM has been observed in association with low RDW, suggesting its potential as a surrogate marker for reduced RBC survival; RDW in this study was calculated as the width of the RBC distribution curve at a relative height of 20% above the baseline [47]. 

In contrast, high RDW levels were reported to be linked with a higher risk and a poor prognosis for diabetic nephropathy and could serve as a tool to assess the influence of therapy [48,49,50]. Increased RDW (CV) was shown to be significantly associated with increased long-term all-cause mortality in DM patients after percutaneous coronary intervention [51]. Changes in both RDW (CV) and MCV showed a positive correlation with HBA1c levels and diabetic retinopathy development and progression [52]. On the other hand, Magri et al. reported a lack of association between RDW (CV), neuropathy, and peripheral artery disease in T2D patients [53]. Despite the potential utility of RDW as a risk assessment tool, RDW may be impacted by preanalytical and analytical phase variables, making it difficult to interpret patient results [11]. Thus, the variations in RBC morphology and size in DM-associated anemia allude to a wide array of underlying mechanisms involved in the pathogenesis of RBC dysfunction, anemia, and DM-related complications. 

## 3. Multifactorial Mechanisms of RBC Dysfunction and Anemia in Diabetes

### 3.1. Nephropathy and Reduced Erythropoiesis in Diabetes-Associated Anemia

The underlying mechanisms of RBC dysfunction and anemia in DM are multifactorial in nature, with reduced erythropoietin (EPO) production being a commonly cited cause of anemia across various glomerular filtration rates. Diminished EPO production is closely associated with the presence of renal microvascular complications and sympathetic denervation of the kidney associated with autonomic neuropathy. In this purview, endogenous EPO production has been suggested as a valid marker of tubulointerstitial damage in DM [54]. Another possible mechanism that could contribute to reduced EPO expression is reduced stability and enhanced degradation of hypoxia-inducible factor-1α because of chronic hyperglycemia and increased ROS levels [55,56]. This mechanism, in turn, could decrease the capacity of renal tubular cells to adapt to hypoxia. 

Additionally, renal failure per se contributes to decreased EPO production, leading to anemia. The presence of diabetes predisposes individuals to systemic inflammation, which adversely affects the interstitial tissue of the kidneys, ultimately culminating in anemia. Anemia associated with EPO deficiency can occur early in DM nephropathy before the onset of advanced kidney failure [57]. However, in another study, it was reported that 70% of anemic patients without compromised renal function also had reduced EPO levels [58], suggesting that the likelihood of functional EPO deficiency related to anemia is independent of the severity of renal failure [59,60].

The presence of overt anemia in DM patients is enhanced with concurrent renal failure and/or increased albuminuria [61]. Results from the Prevalence of Anemia in Early Renal Insufficiency (PAERI) study documented a higher incidence of anemia in DM patients with chronic kidney disease as compared to non-DM patients with chronic kidney disease [62]. These findings substantiate prior observations showing increased severity of anemia in non-dialyzed DM patients with renal failure [63]. While proteinuria has been shown to contribute to the development of anemia in mouse models of nephrotic syndrome without DM [64,65], there are a limited number of reports linking anemia with proteinuria in humans [66]. In DM patients, however, no concrete clinical evidence is available that mechanistically links proteinuria with anemia. Remarkably, a recent study found a significant prevalence of anemia unrelated to renal failure, both in patients with DM and pre-DM, suggesting that compromised renal function is one of several players in the pathogenesis of anemia in DM patients [35]. 

A reduced response of the bone marrow to EPO stimulation may also contribute to diminished erythropoiesis in DM patients. This appears to be a plausible explanation since microvascular damage in DM is likely to impact perfusion to the bone marrow as well as abnormal levels of cytokines related to widespread inflammation in DM [61,67,68]. Notably, some evidence shows that the accumulation of advanced glycation end products (AGEs) in DM can also influence hematopoiesis in the bone marrow [69]. Whether or not this mechanism directly affects RBC progenitors is unknown. 

### 3.2. Iron Deficiency in Diabetes-Associated Anemia

Iron is an essential component of biological processes, such as oxygen delivery, inflammatory response, and erythropoiesis [70,71]. In the heme proteins, iron is an integral component to facilitate oxygen delivery and storage [72]. Ferritin sequesters iron for storage and is an indicator of the body’s iron status [72]. In the guideline evaluation of iron deficiency by Ioannou et al., a parameter of the diagnosis of iron deficiency anemia should include serum ferritin levels of <15–25 ng/mL [73]. The crosstalk between DM and iron deficiency can be garnered from reduced ferritin levels being associated with an increase in RBC lifespan and HbA1c [74,75]. An increase in circulating RBC age can contribute to high HbA1c levels, as seen in patients with DM [75]. Among patients with T2D, iron deficiency anemia may misrepresent the glycemic status of patients due to elevated HbA1c levels [75]. Moreover, high HbA1c levels due to iron deficiency anemia can occur despite plasma glucose levels being controlled [75]. In patients with DM and end-stage renal disease, iron deficiency was found to be relatively common [76]. This observation can be logically comprehended with the consideration of chronic hyperglycemia triggering the modulation of transferring receptors by glycation. This may reduce binding rates of these receptors to iron and thus attenuate iron availability [77]. Patients with DM and chronic renal failure have an increased risk of developing iron deficiency anemia as inflammation induced by chronic kidney disease impedes intestinal iron absorption [78]. 

### 3.3. Role of Inflammation in Diabetes-Associated Anemia

Since DM is characterized by a chronic inflammatory state, it is a plausible notion to consider chronic inflammation as a pivotal mechanism in the pathogenesis of RBC dysfunction and anemia in DM patients. Upregulation of various proinflammatory cytokines, such as interleukin-1, tumor-necrosis factor α, and interferon-γ, have been mechanistically implicated in the development of anemia in various chronic clinical conditions [22,68,79,80]. Typical features of hematological changes related to anemia of inflammation include the presence of normochromic and normocytic anemia with a shortened RBC lifespan and reduced erythropoiesis [81]. A cardinal feature of inflammation-associated anemia is dysregulated iron homeostasis [68]. Systemic immune activation favors profound alterations in various processes, such as iron trafficking, which results in iron accumulation in macrophages and decreases dietary iron absorption [68]. Accordingly, increased hepcidin levels have been documented to promote anemia related to chronic inflammation [82]. More recently, the role of high mobility group box-1 protein, a damage-associated molecular pattern molecule that can affect erythroid precursors essential to normal erythropoiesis, has been demonstrated in anemia stemming from inflammation [83]. 

### 3.4. Testosterone Deficiency as a Potential Link between Anemia and Diabetes

It has long been believed that low testosterone could be a risk factor or a possible cause of anemia in males [84]. Previous cross-sectional studies supported this conclusion having found that testosterone deficiency was significantly associated with anemia [85,86]. Indeed, animal experimentation revealed that testosterone enhances erythropoiesis, at least partly via stimulating EPO production [87]. Surprisingly, testosterone was shown to increase susceptibility to hemolysis in mice and humans [88]. In the context of DM, results from a cross-sectional study showed that testosterone deficiency was associated with an increased frequency of anemia in men with T2D, suggesting possible mechanistic crosstalk between anemia and testosterone deficiency in DM [89]. 

## 4. Multifactorial Mechanisms Explaining Shortened RBC Lifespan in Diabetes

### 4.1. Regulation of RBC Lifespan in Physiologic and Pathologic Conditions

Mounting evidence has shown that RBC survival in the circulation of patients with DM is significantly shortened. This may be a direct consequence of disturbances in the hematological milieu, including extracellular hypertonicity, oxidative stress, and chronic hyperglycemia [22]. These cell stressors affect RBCs, leading to their dysfunction and potentially contributing to the pathophysiology of DM complications [22]. 

Fully developed RBCs traverse the circulatory circuit for a 100- to 120-day period, following their differentiation from erythroblastic progenitors. These progenitors were once derived from the myeloid stem cell lineage within the confines of the bone marrow [90,91,92]. Prior to their maturation, RBCs extrude their nuclei and enter the reticulocyte stage for 1–2 days and contain ribosomal RNA [93]. Fully mature RBCs are anucleate and can accommodate high levels of hemoglobin, thereby maximizing their oxygen-carrying capacity [94]. Physiologic RBC aging is characterized by changes in density, volume, and shape; these occur in parallel to quantitative and qualitative alteration on their surface. Notable changes underlining the aging process of circulating RBCs include clustering of Band 3 protein, complementing C3 deposition, and autologous binding of antibodies to Band 3 [93,94,95]. These alterations promote RBC senescence through the generation of microvesicles and exposure of senescence-specific RBC antigens [93]. Furthermore, changes in glycoprotein moieties, surface CD47 expression, and the architecture of the phospholipid bilayer of the membrane prime the RBCs for removal by phagocytes of the reticuloendothelial system [93]. 

Within circulation, RBCs are exposed to varying physiologic and pathologic environments from which they accrue damages leading to the activation of various ion channels and intracellular enzymes [96,97]. These changes, in turn, cascade into alterations of the phospholipid pattern of the cell membrane, leading to flipping of the aminophospholipid phosphatidylserine [98] on the membrane’s surface [99,100,101]. Collectively, the cellular alterations lead to RBC cell death, also referred to as eryptosis [99,100]. RBC PS can be directly recognized by a host of macrophage receptors, such as Stabilin-2, BAI1, Tim-1, Tim-4, and CD300. PS recognition by macrophages is also facilitated by other receptors, such as integrins αvβ3 and αvβ5 and the Mer tyrosine kinase receptor through soluble bridging PS-binding proteins, including MFG-E8, Gas 6, and protein S [102]. The phagocytosed RBCs are catabolized in the macrophages, with individual components of the RBCs subjected to recycling. 

### 4.2. Putative Intracellular Pathways of Cell Death Signaling in RBCs

With the absence of organelles involved in the apoptosis of nucleated cells, a host of signaling mechanisms orchestrating cell death in RBCs has been identified. These mechanisms become activated when the cell is subjected to stress, such as hyperthermia, glucose starvation, extracellular hypertonicity, and oxidative stress [102,103]. The cell stressors activate Ca^2+^ permeable channels leading to an influx of extracellular Ca^2+^ into the cytoplasm. Increases in intracellular Ca^2+^ levels activate scramblases, leading to externalization of PS on the cell surface [102,103]. In addition, another putative effect of increased cytoplasmic Ca^2+^ is the activation of the K^+^ Gardos channels, which consequently induces water leakage and cell volume reduction [98,102,103]. Enhanced cytoplasmic Ca^2+^ further mediates the activation of a host of other enzymes involved in the RBC’s cell death machinery, such as transglutaminase, phospholipases, calpain, protein kinases, and phosphatases. RBCs can also undergo cell death by mechanisms independent of Ca^2+^ increases, such as ceramide formation and altered nitric oxide (NO) signaling [102,104,105,106]. Cell death signaling triggered by increased extracellular hypertonicity involves p38 mitogen-activated protein kinases (MAPK), mitogen- and stress-activated kinases MSK1/2, and the heterotrimeric G-protein subunit Gαi2 [99,102]. When RBCs are starved of energy, protein kinase C, and the energy-sensing enzyme AMP-activated kinase, AMP-activated kinase (AMPK)α1 regulate cellular death. Another putative signaling pathway promoting RBC cell death involves the interaction between the Ca^2+^-binding trimeric glycoprotein thrombospondin-1 and RBC CD47 [107]. 

### 4.3. Mechanisms of Reduced Lifespan of RBCs in Diabetes

Numerous investigations have provided evidence indicating that RBCs in individuals with DM display heightened vulnerability to damage and experience a reduced lifespan within the circulatory system due to substantial modifications occurring in the extracellular environment. A wide array of putative mechanisms underlying the reduced survival of RBCs in the diabetic milieu have been characterized in recent years. Kempe-Teufel et al. showed that patients with T2D were anemic with reduced hemoglobin and RBC count and displayed a significantly higher proportion of PS-exposing RBCs in circulation [108]. In the same study, RBCs from T2D patients also increased ceramide abundance and ROS levels without significant differences in intracellular Ca^2+^ levels and MCV as compared to RBCs drawn from healthy volunteers [108]. An alternative study found DM patients to have higher PS exposure due to the suppression of flippase enzyme activity, the suppression of which was caused by higher levels of membrane tubulin [109]. RBC cell death in T2D patients was also documented and associated to be with increased RBC caspase 3 activation [110,111]. 

Another potential mechanism underlying enhanced RBC cell death could involve enhanced thrombospondin-1 levels in DM [112,113], as its interactions with RBC CD47 trigger PS externalization [107]. DM patients also display elevated phosphatidic acid and phospholipase D activities, which were shown to elicit RBC PS externalization [114]. DM patients further display reduced NO production in RBCs which is paralleled by increased PS externalization [115]. Remarkably, serum levels of nitrogen-related metabolites regulated by RBCs are disrupted in T2D, as RBCs not only facilitate their transport but also play a critical role in arginine metabolism [116]. Animal studies have shown that long-term and low doses of nitrate had beneficial effects against anemia in obese T2D rats [117]. Increased RBC cell death in DM patients was also shown to be paralleled with increased microparticle release [118]. Animal studies have further confirmed the presence of an increased percentage of PS-exposing RBCs in circulation, supporting the idea that chronic hyperglycemia can confer a pro-apoptotic phenotype in RBCs across other mammalian species [119,120,121]. 

### 4.4. Influence of Concurrent Systemic Conditions on RBC Survival in Diabetes

In some patients, DM may be concomitantly present with other systemic conditions, which are associated with accelerated RBC death within the circulation. Metabolic syndrome, a collection of clinical entities encompassing obesity, hypercholesterolemia, and hypertension can impact RBC viability [97]. When isolated, studies report that these conditions enhance PS externalization and thus result in reduced RBC survival. In a previous study, RBC PS exposure was shown to be significantly higher in individuals with a higher body mass index as compared to subjects with a normal body mass index [122]. Moreover, the expression of the RBC senescence marker CD47 was reported to be significantly reduced in obese patients [123]. Interestingly, RBC dysfunction and membrane PS exposure were shown to be significantly augmented in mice fed with a high-fat diet for a prolonged period, suggesting that obesity in tandem with DM in patients could potentially further reduce RBC survival as compared to patients with isolated DM [124]. 

Accordingly, similar changes in the RBC phenotype of DM patients can be surmised in concomitant conditions such as hypertension and patients with dyslipidemia [109,125,126]. Chronic renal failure can aggravate RBC cell death in DM, as uremia and proteinuria have been shown to reduce RBC survival in multiple studies [64,127,128,129,130,131,132]. Dialysis treatment may further impact RBC survival within DM patients [130,133,134]. In a previous study, chronic kidney disease was shown to enhance oxidative imbalance and PS exposure in RBCs drawn from T2D patients [135]. Strikingly, morphological alterations in RBCs in DM patients may influence the quality of donated blood for transfusion. A recent study showed that donated RBCs from individuals with high HbA1c levels showed increased hemolysis rate, K^+^ efflux, oxidative stress, and PS exposure, characteristic features of the RBC storage lesion [136].

### 4.5. Impact of In Vitro Glycation on RBC Damage 

Experiments using artificial glycation of RBCs by treatment with glucose in vitro have been used to elucidate mechanisms of RBC damage and cell death. Sustained glycation in vitro, simulating the in vivo environment in DM, enhanced HbA1c levels and PS exposure in RBCs [16,137]. High glucose treatment-induced PS externalization, protein glycation, and lipid peroxidation in RBCs were shown to be inhibited by ferulic acid treatment [138]. Prior antioxidant treatment of RBCs was reported to attenuate RBC PS exposure after incubation in a high glucose medium [139]. Notably, the in vitro treatment of RBCs with high glucose concentration was reported to significantly increase the glycation of membrane proteins, TRPC3/6/7, and L-type Ca^2+^ channel proteins, and augment amiloride-sensitive, voltage-independent cation conductance. This leads to increased cytosolic Ca^2+^ and PS externalization, indicating the possible impact of glycation on ion channels involved in RBC cell death [140]. 

### 4.6. Dicarbonyl Stress and Increased Endogenous Toxin Levels in Diabetes

Increased generation of the dicarbonyl compound methylglyoxal (MG) in DM has been documented to trigger cellular dysfunction in DM [141,142,143]. High levels of MG, associated with DM, can occur both in RBCs and plasma [144]. Elevated plasma MG levels have been shown to increase the risk of cardiovascular events in patients with T1D [145]. MG has a potent glycating activity, and its formation occurs via various mechanisms, including protein and nucleic acid modifications, as well as a glycolysis by-product and glucose autoxidation [141,146]. The glyoxalase system detoxifies MG by converting it to D-lactate but if MG levels overwhelm the glyoxalase systems, MG may modify arginine residues on proteins and produce AGEs [141]. A key MG-derived AGE is methylglyoxal-derived-hydroimidazalone-1, which accounts for most of all MG adducts [147]. MG was shown to induce a wide range of effects in RBCs, such as enhanced membrane fragility leading to hemolysis, depletion of amino groups, and increased PS exposure [148,149,150]. MG affects RBCs through deranged energy and oxidative balance and can trigger changes in the deformability and elongation of RBCs [149,150]. In addition to increased MG levels in DM, concomitant nephropathy can lead to high levels of extracellular phosphate as well as the accumulation of uremic toxins, such as p-cresol, indoxyl sulfate, and acrolein, which have been reported to promote RBC cell death [151,152,153,154]. Therefore, therapeutic lowering of these toxins could potentially ameliorate RBC dysfunction and anemia in DM-associated nephropathy. 

## 5. Procoagulant RBC Phenotype and Microangiopathy in Diabetes

### 5.1. Implications of RBC Dysfunction for Thrombotic Risk in Diabetes

Recent studies have demonstrated that RBC defects are associated with increased thrombotic risk [155]. RBCs contribute to thrombogenesis via a wide array of mechanisms involving RBC retention within the clot [155]. RBC retention during clot formation and contraction hinges upon the activation of transglutaminase factor XIII, the process of which is mediated through the crosslinking of fibrin α-chains [156,157]. Hypercoagulability in various systemic diseases, including DM, is promoted by the presence of an increased proportion of not only PS-exposing RBCs but also increased levels of circulating RBC-derived microparticles, which express PS [158,159]. PS externalization on the RBC cell membrane serves as a platform for the assembly of the prothrombinase and tenase complexes, which, in turn, stimulate thrombin generation and clotting, thus mediating the procoagulant effects of PS-exposing apoptotic RBCs [102,160,161]. In addition, surface PS exposure also stimulates coagulation factors V and X, thereby mediating a hypercoagulable state [162]. Interestingly, the procoagulant phenotype of PS-exposing RBCs was previously demonstrated in uremic [127,163] and thalassemic [164] patients as well as in a rat model of lead toxicity [165]. 

### 5.2. Increased RBC–Platelet Interactions in Diabetes

Increased RBC–platelet interactions may further explain the concerning factors of RBC-mediated thrombogenesis. In an in vivo model of FeCl_3_-elicited thrombosis, RBCs were reported to proactively recruit platelets and mediate their adhesion to intact ECs of the vascular wall [166]. Remarkably, platelets express the transmembrane CXC chemokine ligand CXCL16, also known as SR-PSOX, which possesses the capacity to bind to PS on the RBC’s surface [80,167]. Furthermore, platelet–RBC interactions may be fostered via the platelet surface glycoprotein CD36, which displays a propensity for binding to PS [168]. More recently, a platelet–RBC interaction was shown to be mediated by FasL/FasR pathway, which triggers PS externalization on RBCs and thereby enhances their procoagulant activity in circulation [169]. Therefore, it may be surmised that increased levels of PS-exposing RBCs, as well as RBC-derived microparticles, can confer a procoagulant phenotype on RBCs and enhance the risk of thrombotic events in DM, thereby contributing to a wide range of microvascular and macrovascular complications [118]. 

### 5.3. RBCs Promote Vascular Endothelial Cell Dysfunction in Diabetes

ECs, akin to platelets, similarly express CXCL16 on their surface, and various studies have concluded that PS-exposing RBCs are increasingly capable of adhering to ECs on the vascular lining [170]. Likewise, CXCL16 on the EC’s surface mediates its adhesion to leukocytes under flow conditions, contributing to the pathogenesis of atherosclerotic lesions [171]. In theory, the adhesion of RBCs to vascular ECs could synergize with enhanced leukocyte–EC interactions and participate in the development of vascular injury leading to thrombosis in DM. In addition to cell surface CXCL16, EC–RBC interactions were shown to be fostered by EC surface CD36 [172] and thrombospondin [173]. In DM, additional mechanisms involving glycation of Band 3 protein and the receptor for AGE are also believed to play a role in increased RBC–EC interactions [174], which, in turn, could lead to vascular inflammation and hyperpermeability [175]. Remarkably, PS-exposing RBCs were shown to underpin the pathophysiology of retinal vein occlusion, a common cause of DM-associated retinopathy leading to loss of vision [176]. Such a mechanism may further underlie microangiopathy, leading to nephropathy in DM, as PS-exposing RBCs were shown to adhere to the vessel wall in a mouse model of renal ischemia/reperfusion injury [177].

A putative mechanism that potentially mediates blockages in the microvasculature is the formation of aggregates consisting of von Willebrand factor fibers and PS-exposing RBCs [177]. Strikingly, enhanced interactions of dysfunctional glycated RBCs with the vascular lining were reported to promote phagocytosis by ECs [178]. This phenomenon may be strongly linked to EC dysfunction, consequently increasing the risk of atherothrombotic plaque rupture encountered in DM. Indeed, RBCs in T2D were recently shown to trigger EC dysfunction and vascular injury by a mechanism involving the downregulation of miR-210 expression [179], as well as elevated arginase 1 and ROS activity in RBCs [180,181]. In view of these studies, RBC dysfunction thus appears to be a crucial player contributing to the pathophysiology of a wide array of DM-associated complications. 

## 6. RBC Biochemical and Biophysical Alterations in Diabetes

### 6.1. Metabolic and Lipid Alterations of RBCs in Diabetes

Chronic hyperglycemia in DM can potentiate several biochemical changes within RBCs affecting redox and energy metabolism, as well as the transport of certain metabolites required for normal physiological functions. In a recent study, metabolomic and lipidomic characterization of RBCs from T1D patients versus healthy controls revealed significant differences in amino acids, creatine and phosphocreatine, lipid chain length, and choline derivatives, suggesting changes in glycolysis as well as branched-chain amino acid and phospholipid metabolism due to T1D [182]. Similarly, metabolic derangements were also reported in the RBCs of T2D patients. The metabolomic profiling of T2D RBCs showed changes in a wide range of metabolites, including 2,3-bisphophoglycerate, inosinic acid, lactate, 6-phosphogluconate, creatine, and adenosine triphosphate [183]. Furthermore, the study also reported changes in a host of amino acids, such as leucine, glycine, alanine, lysine, aspartate, phenylalanine, and tyrosine [183]. Interestingly, RBCs from T2D patients were shown to synthesize glutathione normally despite increased oxidative stress [184]. Membrane compositional alterations in RBCs from T2D patients, gleaned through lipidomic analyses, has revealed an enhanced content of cholesterol, total sphingolipids, sphingomyelin, and glycolipids, and a decreased total of phospholipids [185]. Remarkably, RBCs from T2D patients further revealed that lipids were esterified with saturated rather than unsaturated fatty acids, a mechanism potentially influencing their biophysical properties that is discussed below [185]. Changes in cell membrane lipid profile were further observed in the RBCs of patients with gestational DM, suggesting that DM can alter both cellular metabolism and the lipid composition of the RBC membrane [186].

### 6.2. Alterations in RBC Membrane Properties in Diabetes

Several studies have propounded hemorheological perturbations of RBCs in DM, attributed to changes in their membrane properties [187,188,189,190,191]. Biophysical and constitutional alterations of the RBC plasma membrane in DM affect their physiological deformability, membrane fragility, and aggregability in circulation. RBCs must maintain a balance in these biophysical properties to traverse the microcirculation without impedance [192]. The vulnerability of RBCs to rupture and hemolysis can be furthered through the weakening of the plasma membranes. The progressive membrane microvesiculation of RBCs to eliminate oxidative waste products with concurrent loss of cytoplasmic components ultimately leads to the increased density and rigidity of aged RBCs in circulation [93]. RBC vesiculation has further been linked to hemoglobin loss from RBCs [193]. In the laboratory, RBC deformability is quantified using an ektacytometer, a laser-diffraction viscometer that detects changes in cell water content, surface area, and heterogeneity in these cellular properties [194]. In various systemic diseases, including DM, a myriad of factors dictates RBC deformability changes, such as the physiochemical properties of the extracellular environment, purinergic signaling, oxidative damage, NO, and ionic balance [96]. 

### 6.3. Significance of Hemorheological Changes on Microangiopathic Complications in Diabetes

Changes in hemorheological parameters impact normal oxygen transport to tissues and hence orchestrate functional microangiopathic complications in DM [190,195]. RBCs from DM patients display a host of membrane abnormalities due to various post-translational modifications of membrane properties that directly influence lipid density and fluidity variations in the plasma membrane [196]. These results are supported by recent observations that clarify an impaired elongation index, a quantifiable marker of RBC deformability, following artificial glycation of RBCs in vitro [16]. Clinically, decreased RBC deformability has been speculated to reduce capillary flow in microcirculation, prolonging wound healing in DM patients with foot ulcers. This notion appears to be plausible as a previous study showed a significantly higher percentage of circulating RBCs with reduced deformability in DM patients with food disease versus DM patients with no complications [197]. Although various putative mechanisms regulating RBC deformability changes in DM have been described, the deranged oxidative status of spectrin has also been implicated in RBC deformability changes [198]. 

Another essential determinant of RBC deformability changes ascribed to DM is disturbances in NO bioavailability [199]. DM patients were reported to have an increased proportion of older circulating RBCs and displayed higher NO synthase (NOS) activation and NO production rates in older RBCs to potentially counter the negative effects of cell volume reduction on RBC deformability [199]. Interestingly, endurance training was shown to have beneficial effects on RBC hemorheology in T2D males by increasing RBC deformability in younger RBCs without altering RBC NOS activation [200]. In addition, RBC deformability in DM patients was shown to be altered by the accumulation of sorbitol via the polyol pathway in RBCs due to chronic hyperglycemia [201]. In another study, treatment with the aldose reductase inhibitor sorbinil was demonstrated to attenuate RBC deformability changes in diabetic rats, providing further evidence of the role of sorbitol accumulation in influencing RBC rheology [202]. Accordingly, sorbinil treatment has recently been proposed as a potential therapeutic approach in the management of DM complications [203]. 

### 6.4. Enhanced Hemolysis and RBC Hyperaggregability in Diabetes

Morphology changes in DM patients’ RBCs can also promote redox-sensitive fragility of the cell membrane, leading to hemolysis [16]. Within recent years, patients with T2D appear to have low-grade intravascular hemolysis, alluding to an enhanced breakdown of RBCs in the circulation [204]. The increased heme-related absorbance in these patients promises an association with peripheral sensory neuropathy [204]. RBC creatine, a marker of in vivo hemolysis, was observed to be significantly higher in female T2D patients as compared to healthy subjects [205]. In addition to alterations in deformability and membrane fragility, RBCs in DM patients display hyperaggregability due to changes in cell–cell adhesion dynamics [206,207]. In DM, RBCs are prone to fibrinogen-dependent linear stacking and the formation of a chain-like pattern called roleau, eliciting blood viscosity changes within capillaries [208]. Importantly, the hyperaggregability of RBCs in DM may directly contribute to impaired tissue oxygenation and disturbed organ function. In T2D patients’ association between a quantifiable parameter of RBC aggregability, also known as higher critical shear stress, and reduced deformability with impaired kidney function were found, thus suggesting the pivotal role of RBC dysfunction in triggering complications of DM [209,210].

## 7. Conclusions and Future Directions

**Figure 1:**
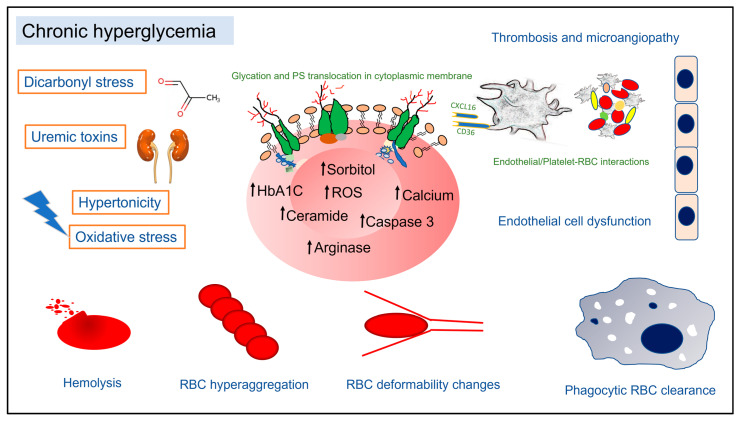
Overview of causes and consequences of RBC dysfunction in diabetes.

Overall, regardless of the confounding etiology, DM patients display RBC alterations (summarized in Figure 1), which may manifest as anemia in a significant proportion of patients. These alterations are especially relevant in the pathophysiology of DM complications, which not only cause RBC dysfunction but are a direct consequence of changes in the biochemical and biophysical properties of RBCs affecting their biorheology and interactions with platelets and the vascular endothelium. Further studies are required to ascertain the mechanisms of these cell–cell interactions, the signal transduction regulating altered RBC metabolism and cell death, as well as their biorheology. Future directions of research in RBC physiology using “omics” technologies in DM are expected to provide new insights into alterations in the RBC proteome, lipids, and metabolites, potentially serving as novel biomarkers to gauge disease progression. It will be of interest to better understand these molecular changes in the context of RBC functions and biophysical properties and their possible linkages to the development of pathological microvascular changes. Since DM patients are known to display altered immune responses, a mechanistic understanding of RBC interactions with immune cells and their phagocytosis would shed more light on RBC survival patterns in the circulation and the pathogenesis of anemia. Based on the current evidence from human and animal studies, future research into the pharmacological targeting of RBC dysfunction in alleviating DM-related microvascular complications will facilitate reducing increased morbidity and mortality associated with DM. 

## Data Availability

Not applicable.
